# Synthesis and crystal structures of 3-hy­droxy-2,4-dimethyl-2*H*-thio­phen-5-one and 3-hy­droxy-4-methyl-2*H*-thio­phen-5-one

**DOI:** 10.1107/S2056989020008269

**Published:** 2020-06-30

**Authors:** Asma Nashawi, Christopher P. Lawson, M. Omar Abdel-Sattar, Joop H. ter Horst, Geoffrey D. Coxon, Alan R. Kennedy

**Affiliations:** a Strathclyde Institute for Pharmacy and Biomedical Sciences, University of Strathclyde, 161 Cathedral Street, Glasgow G4 0RE, Scotland; b Department of Pharmaceutical Chemistry, King Abdul Aziz University, Jeddah, 21589, Saudi Arabia; c Department of Chemistry, Al-Azhar University, Cairo, 11884, Egypt; d EPSRC Future Manufacturing Research Hub for Continuous Manufacture and Advanced Crystallisation (CMAC), Technology and Innovation Centre, University of Strathclyde, 99 George Street, Glasgow G1 1RD, Scotland; e Westchem, Department of Pure & Applied Chemistry, University of Strathclyde, 295 Cathedral Street, Glasgow G1 1XL, Scotland

**Keywords:** crystal structure, hy­droxy-thio­phenone, hydrogen bonding

## Abstract

The structures of two hy­droxy-thio­phenone derivatives related to the anti­biotic thiol­actomycin are presented. These are the racemic 3-hy­droxy-2,4-dimethyl-2*H*-thio­phen-5-one, C_6_H_8_O_2_S, and 3-hy­droxy-4-methyl-2*H*-thio­phen-5-one, C_5_H_6_O_2_S. The main structural feature of both compounds is *C*(6) hydrogen-bonded chains formed between the OH and C=O groups. In achiral C_5_H_6_O_2_S, these chains propagate only by translation, corresponding to *x* + 1, *y*, *z* + 1. However, in contrast, for racemic C_6_H_8_O_2_S the hydrogen-bonded chains propagate through a −*x* + , *y* + , *z* operation, giving chains lying parallel to the crystallographic *b*-axis direction that are composed of alternate *R* and S enanti­omers. The crystals of 3-hydroxy-4-methyl-2H-thiophen-5-one were found to be twinned by a 180° rotation about the reciprocal 001 direction. In the final refinement the twin ratio refined to 0.568 (2):0.432 (2).

## Abstract 2

The structures of two hy­droxy-thio­phenone derivatives related to the anti­biotic thiol­actomycin are presented. The main structural feature of both compounds is *C*(6) hydrogen-bonded chains formed between the OH and C=O groups.

## Chemical context   

Thiol­actomycin (TLM) 1, (5*R*)-4-hy­droxy-3,5-dimethyl-5-[(1*E*)-2-methyl­buta-1,3-dienyl]­thio­phen-2-one, is a naturally occurring anti­biotic isolated from *Norcardia spp* (Sasaki *et al.*, 1982 ▸). Over the last three decades, synthetic efforts towards the synthesis of the single enanti­omer and analogues have provided relatively complex solutions. These have exploited asymmetric synthesis, diasteromeric recrystallization or enzymatic resolution requiring between seven and eleven steps and thus have significantly restricted the development of this scaffold towards clinical application. For examples see Chambers & Thomas (1997 ▸), McFadden *et al.* (2002 ▸), Ohata & Terashima (2009 ▸), Kamal *et al.* (2008 ▸), Toyama *et al.* (2006 ▸) and Bommineni *et al.* (2016 ▸). Herein we present findings from our initial studies focused on the single-crystal determination of thiol­actone analogues.
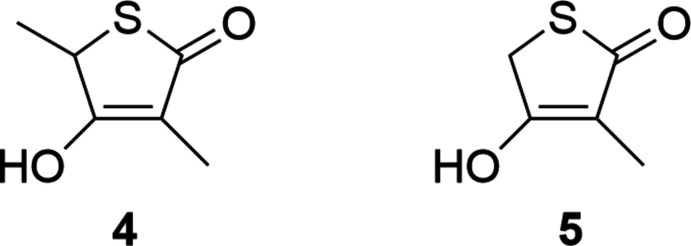


## Structural commentary   

**Figure 1:**
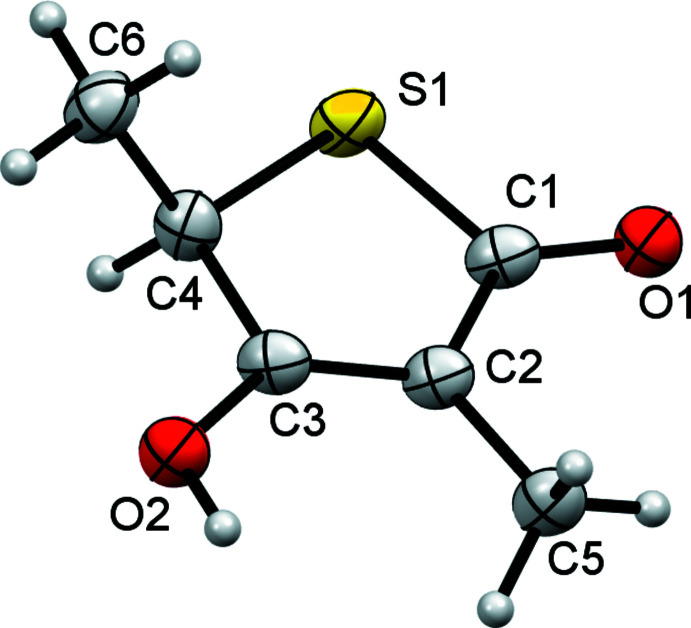
The mol­ecular structure of **4** with non-H atoms shown as 50% probability ellipsoids. H atoms are drawn as small spheres of arbitrary size.

The mol­ecular structures of compounds **4** and **5** are shown in Figs. 1 ▸ and 2 ▸, respectively. As can be seen from Tables 1 ▸ and 2 ▸, equivalent geometric parameters in the two structures are similar, with the largest difference in bond length being found for the S1—C4 values [1.816 (3) and 1.799 (3) Å]. A notable structural difference is the orientation of the hy­droxy groups containing the O2 atoms. In both structures, this O atom is coplanar with the SC_4_ ring, but in structure **4** the H atom points towards C5 and is eclipsed by the C2=C3 double bond whilst in structure **5** the H atom points towards the CH_2_ group and is eclipsed by the C3—C4 single bond. This change in orientation is associated with a change in the bond angles involving O2 [compare C4—C3—O2 angles of 113.4 (2) and 119.7 (3)°]. A search of the Cambridge Structural Database (version 5.40; Groom *et al.*, 2016 ▸) found only three other structures with similar 4-hy­droxy-thio­phen-2-one cores. These are TLM itself (BIHKIM, Nawata *et al.*, 1989 ▸) and two other derivatives (FIVKEA, Chambers *et al.*, 1987 ▸; POXZOS, Kikionis *et al.*, 2009 ▸). These have generally similar geometric parameters to those of **4** and **5**. Two of the database structures have the same hy­droxy group orientation as **5**. Only POXZOS has the same hy­droxy orientation as **4**, and here this orientation is predetermined by the OH group participating in an intra­molecular six-membered hydrogen-bonded ring. As with structures **4** and **5**, in the database structures the orientation of the OH group is associated with systematic changes to the C—C—O bond angles involving OH.

## Supra­molecular features   

**Figure 3:**
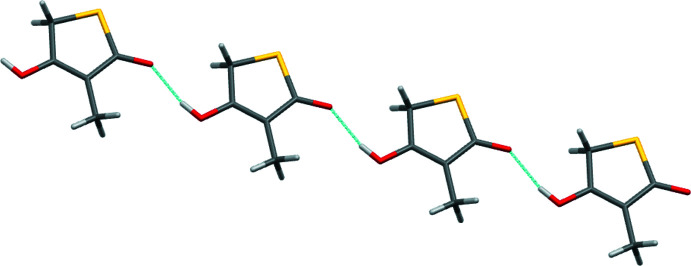
Part of the hydrogen-bonded chain motif present in the structure of **5**. The chain extends parallel to the [101] direction.

**Figure 4:**
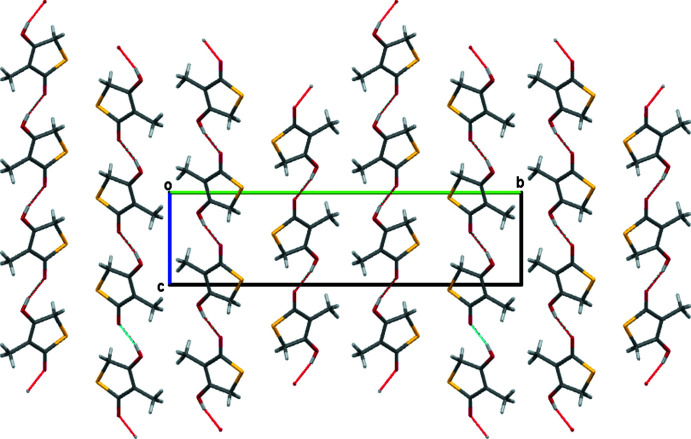
Packing diagram for compound **5** in a view down the *a* axis. Note the alternating Me⋯Me and S⋯S layers.

**Figure 5:**
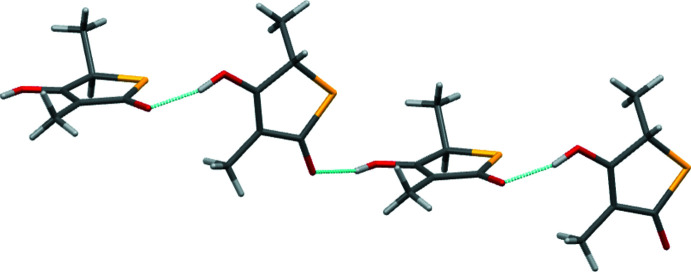
Part of the hydrogen-bonded chain motif present in the structure of **4**. The chain extends parallel to the cystallographic *b* axis.

**Figure 6:**
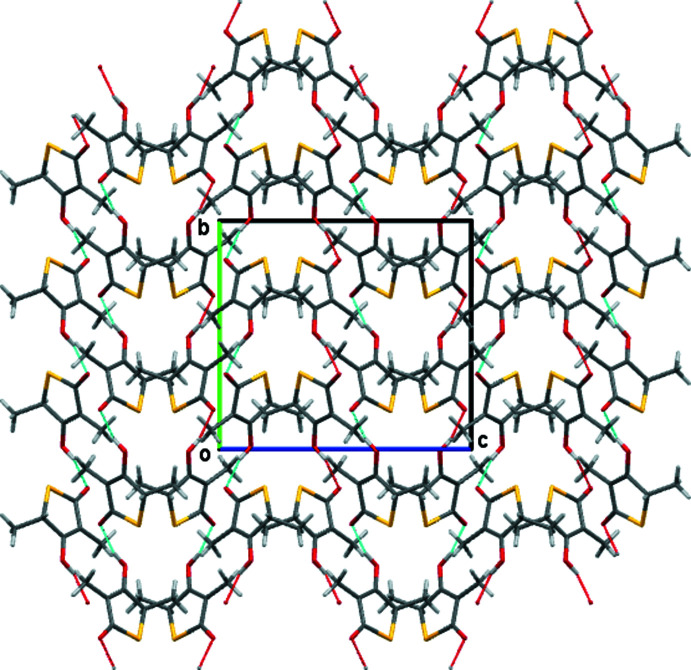
Packing diagram for compound **4** with a view down the *a* axis.

The main supra­molecular feature of both structures **4** and **5** is a one-dimensional *C*(6) hydrogen-bonded chain utilizing OH as the donor group and O1 as the acceptor group, see Tables 3 ▸ and 4 ▸. Behind these basic similarities there lies a great deal of difference in detail. In **5** the chains propagate by translations corresponding to *x* + 1, *y*, *z* + 1. This propagation by translation alone gives the repeating pattern shown in Fig. 3 ▸ where all of the SC_4_ rings of the hydrogen-bonded unit are coplanar and all of the S atoms lie on the same side of the chain. When travelling along the *b*-axis direction, neighbouring chains bear their S atoms on different sides, giving the layered structure shown in Fig. 4 ▸. In contrast, for structure **4** the chain propagates through a −*x* + 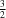, *y* + 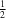, *z* operation giving a chain lying parallel to the crystallographic *b*-axis direction. As shown in Fig. 5 ▸, this results in the neighbouring *R* and S enanti­omers of the racemic chain having perpendicular relationships between the planes of their SC_4_ rings. This different chain geometry gives a very different packing arrangement from that of structure **5**, see Fig. 6 ▸. The only inter­chain contact significantly shorter than the sum of van der Waals radii in either structure occurs in structure **5**. This is a C—H⋯O contact between the CH_2_ group and the ketone O atom. Of the 4-hy­droxy-thio­phen-2-one structures described in the literature, both those of FIVKEA and BIHKIM (TLM) display the same *C*(6) hydrogen-bonded chain motif as **4** and **5**. In both cases, the geometrical detail of the chain is similar to that found in **4**, with the difference that both literature examples are enanti­opure. The final structure, POXZOS, contains additional carb­oxy­lic acid and carbonyl groups and these strong hydrogen-bonding groups dominate the inter­molecular contacts formed and so stop the formation of the otherwise common *C*(6) motif.

## Synthesis and crystallization   

**General synthesis of thiol­actone analogues 4 and 5:** 
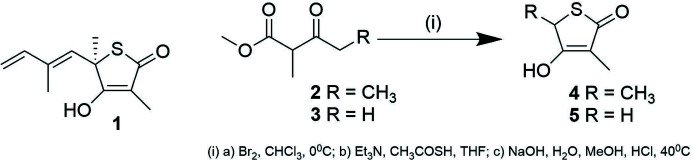


Bromine (1.1 eq) was added to a stirring solution of the corresponding oxoester (1 eq.) dissolved in chloro­form (50 ml) at 273 K. The mixtures were allowed to warm to ambient temperature and stirred for 20 h before removing the solvent under vacuum. The resulting crude mixtures were dissolved in THF (50 ml) before adding tri­methyl­amine (1.1 eq.) and thio­acetic acid (1.1 eq.) and stirring at ambient temperature for a further 18 h. The resulting mixtures were reduced under vacuum to give dark-orange oils that were vacuum filtered over silica using petrol (40/60) and diethyl ether (5:2) as eluent before removing the solvent. The mixtures were dissolved in ethanol (50 ml) before adding a solution of sodium hydroxide (2 eq.) dissolved in water (20 ml) and stirring for 24 h at 333 K. After cooling, HCl (0.1*M*) was added until the solutions reached pH 5 before washing with ethyl acetate (3 × 50 ml) and drying over anhydrous magnesium sulfate. The mixtures were reduced under vacuum and precipitated using petrol (40/60) and diethyl ether to give the products as a solid. For **4**, crystals suitable for crystallographic analysis were grown from a THF solution. For **5**, crystals were grown from a toluene solution.

**3-Hy­droxy-2,4-dimethyl-2H-thio­phen-5-one (4)** Off-white solid (1.1 g, 22%): m.p. 408–409 K. ^1^H NMR (CDCl_3_) δ 4.12 (*dd*, *J* = 7.1, 1.3 Hz, 1H), 1.57 (*d*, *J* = 7.1 Hz, 4H). ^13^C NMR (CDCl_3_) δ 197.16, 177.63, 111.32, 77.26, 77.21, 77.00, 76.75, 42.99, 18.80, 7.62.

**3-Hy­droxy-4-methyl-2** ***H*** **-thio­phen-5-one (5)**

Off-white solid (1.6 g, 18%): m.p. 397–398 K. ^1^H NMR (CDCl_3_) δ 3.94 (*s*, 2H), 1.68 (*s*, 3H). ^13^C NMR (CDCl_3_) δ 195.1, 175.2, 111.2, 32.1, 7.2.

## Refinement   

Crystal data, data collection and structure refinement details are summarized in Table 5 ▸. For both structures, C-bound H atoms were placed in the expected geometric positions and treated in riding modes with C—H = 0.98, 0.99 and 1.00 Å for methyl, CH_2_ and CH groups, respectively. *U*_iso_(H) = 1.5*U*_eq_(C) for methyl groups and 1.2*U*_eq_(C) for the other CH groups. The H atoms of the hy­droxy groups were refined isotropically. Data collection on **5** was carried out by the National Crystallography Service (Cole & Gale, 2012 ▸). The crystals of **5** were found to be twinned by a 180° rotation about the recip­rocal 001 direction. This feature was accounted for by producing a hklf 5 formatted datafile during data processing. In the final refinement the twin ratio refined to 0.568 (2):0.432 (2).

## Supplementary Material

Crystal structure: contains datablock(s) 4, 5, global. DOI: 10.1107/S2056989020008269/lh5954sup1.cif

Structure factors: contains datablock(s) 4. DOI: 10.1107/S2056989020008269/lh59544sup2.hkl

Structure factors: contains datablock(s) 5. DOI: 10.1107/S2056989020008269/lh59545sup3.hkl

Supporting information file. DOI: 10.1107/S2056989020008269/lh59544sup4.cml

Supporting information file. DOI: 10.1107/S2056989020008269/lh59545sup5.cml

CCDC references: 2011283, 2011284

Additional supporting information:  crystallographic information; 3D view; checkCIF report

## Figures and Tables

**Figure 2:**
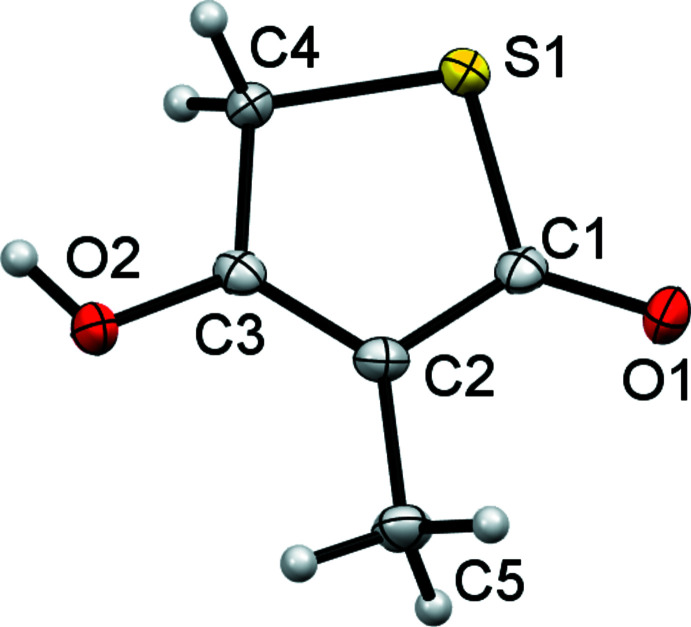
The mol­ecular structure of **5** with non-H atoms shown as 50% probability ellipsoids. H atoms are drawn as small spheres of arbitrary size.

**Table 1:** Selected geometric parameters (Å, °) for **4** [Chem scheme1]

S1—C1	1.778 (3)	C3—C2	1.351 (4)
S1—C4	1.816 (3)	C3—C4	1.507 (4)
O1—C1	1.238 (3)	C2—C1	1.434 (4)
O2—C3	1.326 (3)		
			
C1—S1—C4	92.62 (12)	C3—C2—C1	112.3 (2)
O2—C3—C4	113.4 (2)	C2—C1—S1	112.1 (2)
C2—C3—C4	117.8 (2)	C3—C4—S1	105.16 (18)

**Table 2:** Selected geometric parameters (Å, °) for **5** [Chem scheme1]

S1—C1	1.775 (3)	C1—C2	1.440 (4)
S1—C4	1.799 (3)	C2—C3	1.347 (4)
O1—C1	1.231 (4)	C2—C5	1.502 (4)
O2—C3	1.332 (4)	C3—C4	1.499 (4)
			
C1—S1—C4	92.33 (15)	O2—C3—C4	119.7 (3)
C2—C1—S1	112.0 (2)	C2—C3—C4	117.2 (3)
C3—C2—C1	112.2 (3)	C3—C4—S1	106.2 (2)

**Table 3:** Hydrogen-bond geometry (Å, °) for **4** [Chem scheme1]

*D*—H⋯*A*	*D*—H	H⋯*A*	*D*⋯*A*	*D*—H⋯*A*
O2—H1*H*⋯O1^i^	0.88 (4)	1.77 (4)	2.621 (3)	164 (4)

Symmetry code: (i) 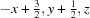.

**Table 4:** Hydrogen-bond geometry (Å, °) for **5** [Chem scheme1]

*D*—H⋯*A*	*D*—H	H⋯*A*	*D*⋯*A*	*D*—H⋯*A*
O2—H1*H*⋯O1^i^	0.84 (5)	1.80 (5)	2.629 (3)	168 (5)
C4—H4*B*⋯O1^ii^	0.99	2.58	3.552 (4)	169

Symmetry codes: (i) 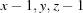; (ii) 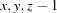.

**Table 5:** Experimental details

	**4**	**5**
Crystal data		
Chemical formula	C_6_H_8_O_2_S	C_5_H_6_O_2_S
*M*_r_	144.18	130.16
Crystal system, space group	Orthorhombic, *P* *b* *c* *a*	Monoclinic, *P*2_1_/*c*
Temperature (K)	123	100
*a*, *b*, *c* (Å)	9.286 (1), 11.4809 (8), 12.6469 (10)	4.1054 (3), 22.9727 (13), 6.1928 (5)
α, β, γ (°)	90, 90, 90	90, 103.728 (7), 90
*V* (Å^3^)	1348.3 (2)	567.37 (7)
*Z*	8	4
Radiation type	Cu *K*α	Mo *K*α
μ (mm^−1^)	3.63	0.46
Crystal size (mm)	0.55 × 0.08 × 0.04	0.12 × 0.02 × 0.01

Data collection		
Diffractometer	Oxford Diffraction Gemini S	Rigaku XtaLAB AFC12
Absorption correction	Analytical (*CrysAlis PRO*; Rigaku OD, 2019 ▸)	Multi-scan (*CrysAlis PRO*; Rigaku OD, 2019 ▸)
*T*_min_, *T*_max_	0.323, 0.847	0.766, 1.000
No. of measured, independent and observed [*I* > 2σ(*I*)] reflections	4293, 1323, 1115	2121, 2121, 1955
*R*_int_	0.049	0.026
(sin θ/λ)_max_ (Å^−1^)	0.620	0.650

Refinement		
*R*[*F* ^2^ > 2σ(*F* ^2^)], *wR*(*F* ^2^), *S*	0.053, 0.148, 1.07	0.050, 0.113, 1.19
No. of reflections	1323	2121
No. of parameters	88	79
H-atom treatment	H atoms treated by a mixture of independent and constrained refinement	H atoms treated by a mixture of independent and constrained refinement
Δρ_max_, Δρ_min_ (e Å^−3^)	0.67, −0.29	0.43, −0.33

Computer programs: *CrysAlis PRO* (Rigaku OD, 2019 ▸), *SIR92* (Altomare *et al.*, 1994 ▸), *SHELXL2014* (Sheldrick, 2015 ▸), *Mercury* (Macrae *et al.*, 2020 ▸) and *ORTEP-3 for Windows* (Farrugia, 2012 ▸).

## supplementary crystallographic information

### 3-Hydroxy-2,4-dimethyl-2H-thiophen-5-one (4) Crystal data

**Table:**

C_6_H_8_O_2_S	*D*_x_ = 1.421 Mg m^−^^3^
*M**_r_* = 144.18	Cu *K*α radiation, λ = 1.54184 Å
Orthorhombic, *P**b**c**a*	Cell parameters from 1393 reflections
*a* = 9.286 (1) Å	θ = 7.0–72.8°
*b* = 11.4809 (8) Å	µ = 3.63 mm^−^^1^
*c* = 12.6469 (10) Å	*T* = 123 K
*V* = 1348.3 (2) Å^3^	Rod, colourless
*Z* = 8	0.55 × 0.08 × 0.04 mm
*F*(000) = 608	

### 3-Hydroxy-2,4-dimethyl-2H-thiophen-5-one (4) Data collection

**Table:**

Oxford Diffraction Gemini S diffractometer	1115 reflections with *I* > 2σ(*I*)
Radiation source: sealed tube	*R*_int_ = 0.049
ω scans	θ_max_ = 73.0°, θ_min_ = 7.0°
Absorption correction: analytical (CrysAlisPro; Rigaku OD, 2019)	*h* = −11→11
*T*_min_ = 0.323, *T*_max_ = 0.847	*k* = −13→10
4293 measured reflections	*l* = −15→12
1323 independent reflections	

### 3-Hydroxy-2,4-dimethyl-2H-thiophen-5-one (4) Refinement

**Table:**

Refinement on *F*^2^	0 restraints
Least-squares matrix: full	Hydrogen site location: mixed
*R*[*F*^2^ > 2σ(*F*^2^)] = 0.053	H atoms treated by a mixture of independent and constrained refinement
*wR*(*F*^2^) = 0.148	*w* = 1/[σ^2^(*F*_o_^2^) + (0.081*P*)^2^ + 0.8472*P*] where *P* = (*F*_o_^2^ + 2*F*_c_^2^)/3
*S* = 1.07	(Δ/σ)_max_ < 0.001
1323 reflections	Δρ_max_ = 0.67 e Å^−^^3^
88 parameters	Δρ_min_ = −0.29 e Å^−^^3^

### 3-Hydroxy-2,4-dimethyl-2H-thiophen-5-one (4) Special details

**Table:**

Geometry. All esds (except the esd in the dihedral angle between two l.s. planes) are estimated using the full covariance matrix. The cell esds are taken into account individually in the estimation of esds in distances, angles and torsion angles; correlations between esds in cell parameters are only used when they are defined by crystal symmetry. An approximate (isotropic) treatment of cell esds is used for estimating esds involving l.s. planes.

### 3-Hydroxy-2,4-dimethyl-2H-thiophen-5-one (4) Fractional atomic coordinates and isotropic or equivalent isotropic displacement parameters (Å^2^)

**Table:**

	*x*	*y*	*z*	*U*_iso_*/*U*_eq_	
S1	0.84281 (8)	0.16688 (5)	0.67914 (5)	0.0327 (3)	
O1	0.6506 (2)	0.17209 (14)	0.52486 (16)	0.0311 (5)	
O2	0.9490 (2)	0.48890 (16)	0.62458 (16)	0.0329 (5)	
C3	0.8868 (3)	0.3850 (2)	0.6199 (2)	0.0283 (6)	
C6	0.9207 (4)	0.3465 (3)	0.8144 (2)	0.0374 (7)	
H6A	0.9733	0.4199	0.8231	0.056*	
H6B	0.9571	0.2890	0.8651	0.056*	
H6C	0.8179	0.3597	0.8271	0.056*	
C2	0.7822 (3)	0.3476 (2)	0.5539 (2)	0.0272 (6)	
C1	0.7427 (3)	0.2288 (2)	0.57332 (19)	0.0281 (6)	
C5	0.7097 (3)	0.4157 (2)	0.4680 (2)	0.0330 (6)	
H5A	0.6367	0.4670	0.4990	0.049*	
H5B	0.6636	0.3619	0.4182	0.049*	
H5C	0.7815	0.4627	0.4304	0.049*	
C4	0.9423 (3)	0.3008 (2)	0.7017 (2)	0.0302 (6)	
H4	1.0472	0.2864	0.6892	0.036*	
H1H	0.914 (5)	0.542 (3)	0.581 (3)	0.053 (11)*	

### 3-Hydroxy-2,4-dimethyl-2H-thiophen-5-one (4) Atomic displacement parameters (Å^2^)

**Table:**

	*U*^11^	*U*^22^	*U*^33^	*U*^12^	*U*^13^	*U*^23^
S1	0.0477 (5)	0.0172 (4)	0.0332 (4)	0.0017 (2)	−0.0059 (3)	0.0025 (2)
O1	0.0410 (11)	0.0178 (9)	0.0344 (10)	−0.0013 (7)	−0.0046 (7)	−0.0024 (7)
O2	0.0438 (11)	0.0188 (9)	0.0360 (10)	−0.0026 (8)	−0.0046 (8)	−0.0005 (7)
C3	0.0383 (13)	0.0172 (11)	0.0294 (12)	0.0014 (10)	0.0030 (10)	−0.0021 (9)
C6	0.0475 (17)	0.0328 (14)	0.0318 (14)	−0.0009 (12)	−0.0047 (12)	−0.0014 (11)
C2	0.0377 (14)	0.0178 (12)	0.0262 (11)	0.0024 (10)	0.0017 (10)	−0.0022 (9)
C1	0.0389 (14)	0.0187 (12)	0.0266 (11)	0.0046 (10)	0.0025 (10)	−0.0012 (9)
C5	0.0473 (15)	0.0183 (13)	0.0334 (13)	−0.0006 (11)	−0.0048 (11)	0.0004 (9)
C4	0.0352 (13)	0.0208 (12)	0.0346 (13)	0.0014 (10)	−0.0035 (10)	−0.0018 (10)

### 3-Hydroxy-2,4-dimethyl-2H-thiophen-5-one (4) Geometric parameters (Å, º)

**Table:**

S1—C1	1.778 (3)	C6—H6B	0.9800
S1—C4	1.816 (3)	C6—H6C	0.9800
O1—C1	1.238 (3)	C2—C1	1.434 (4)
O2—C3	1.326 (3)	C2—C5	1.498 (4)
O2—H1H	0.88 (4)	C5—H5A	0.9800
C3—C2	1.351 (4)	C5—H5B	0.9800
C3—C4	1.507 (4)	C5—H5C	0.9800
C6—C4	1.532 (4)	C4—H4	1.0000
C6—H6A	0.9800		
			
C1—S1—C4	92.62 (12)	O1—C1—S1	121.6 (2)
C3—O2—H1H	116 (3)	C2—C1—S1	112.1 (2)
O2—C3—C2	128.8 (2)	C2—C5—H5A	109.5
O2—C3—C4	113.4 (2)	C2—C5—H5B	109.5
C2—C3—C4	117.8 (2)	H5A—C5—H5B	109.5
C4—C6—H6A	109.5	C2—C5—H5C	109.5
C4—C6—H6B	109.5	H5A—C5—H5C	109.5
H6A—C6—H6B	109.5	H5B—C5—H5C	109.5
C4—C6—H6C	109.5	C3—C4—C6	112.0 (2)
H6A—C6—H6C	109.5	C3—C4—S1	105.16 (18)
H6B—C6—H6C	109.5	C6—C4—S1	111.7 (2)
C3—C2—C1	112.3 (2)	C3—C4—H4	109.3
C3—C2—C5	127.3 (2)	C6—C4—H4	109.3
C1—C2—C5	120.4 (2)	S1—C4—H4	109.3
O1—C1—C2	126.3 (2)		
			
O2—C3—C2—C1	−180.0 (2)	C4—S1—C1—O1	−179.6 (2)
C4—C3—C2—C1	−0.9 (3)	C4—S1—C1—C2	0.2 (2)
O2—C3—C2—C5	0.2 (5)	O2—C3—C4—C6	58.8 (3)
C4—C3—C2—C5	179.2 (2)	C2—C3—C4—C6	−120.4 (3)
C3—C2—C1—O1	−179.8 (3)	O2—C3—C4—S1	−179.77 (18)
C5—C2—C1—O1	0.0 (4)	C2—C3—C4—S1	1.0 (3)
C3—C2—C1—S1	0.4 (3)	C1—S1—C4—C3	−0.64 (19)
C5—C2—C1—S1	−179.8 (2)	C1—S1—C4—C6	121.0 (2)

### 3-Hydroxy-2,4-dimethyl-2H-thiophen-5-one (4) Hydrogen-bond geometry (Å, º)

**Table:**

*D*—H···*A*	*D*—H	H···*A*	*D*···*A*	*D*—H···*A*
O2—H1*H*···O1^i^	0.88 (4)	1.77 (4)	2.621 (3)	164 (4)

Symmetry code: (i) −*x*+3/2, *y*+1/2, *z*.

### 3-Hydroxy-4-methyl-2H-thiophen-5-one (5) Crystal data

**Table:**

C_5_H_6_O_2_S	*F*(000) = 272
*M**_r_* = 130.16	*D*_x_ = 1.524 Mg m^−^^3^
Monoclinic, *P*2_1_/*c*	Mo *K*α radiation, λ = 0.71073 Å
*a* = 4.1054 (3) Å	Cell parameters from 1968 reflections
*b* = 22.9727 (13) Å	θ = 5.2–31.4°
*c* = 6.1928 (5) Å	µ = 0.46 mm^−^^1^
β = 103.728 (7)°	*T* = 100 K
*V* = 567.37 (7) Å^3^	Needle, colourless
*Z* = 4	0.12 × 0.02 × 0.01 mm

### 3-Hydroxy-4-methyl-2H-thiophen-5-one (5) Data collection

**Table:**

Rigaku XtaLAB AFC12 diffractometer	1955 reflections with *I* > 2σ(*I*)
Radiation source: rotating anode	*R*_int_ = 0.026
ω scans	θ_max_ = 27.5°, θ_min_ = 3.5°
Absorption correction: multi-scan (CrysAlisPro; Rigaku OD, 2019)	*h* = −5→5
*T*_min_ = 0.766, *T*_max_ = 1.000	*k* = −29→29
2121 measured reflections	*l* = −8→8
2121 independent reflections	

### 3-Hydroxy-4-methyl-2H-thiophen-5-one (5) Refinement

**Table:**

Refinement on *F*^2^	0 restraints
Least-squares matrix: full	Hydrogen site location: mixed
*R*[*F*^2^ > 2σ(*F*^2^)] = 0.050	H atoms treated by a mixture of independent and constrained refinement
*wR*(*F*^2^) = 0.113	*w* = 1/[σ^2^(*F*_o_^2^) + (0.0078*P*)^2^ + 1.3568*P*] where *P* = (*F*_o_^2^ + 2*F*_c_^2^)/3
*S* = 1.19	(Δ/σ)_max_ < 0.001
2121 reflections	Δρ_max_ = 0.43 e Å^−^^3^
79 parameters	Δρ_min_ = −0.33 e Å^−^^3^

### 3-Hydroxy-4-methyl-2H-thiophen-5-one (5) Special details

**Table:**

Geometry. All esds (except the esd in the dihedral angle between two l.s. planes) are estimated using the full covariance matrix. The cell esds are taken into account individually in the estimation of esds in distances, angles and torsion angles; correlations between esds in cell parameters are only used when they are defined by crystal symmetry. An approximate (isotropic) treatment of cell esds is used for estimating esds involving l.s. planes.
Refinement. Refined as a 2-component twin against a hklf 5 formatted datafile.This datafile was created by CrysalisPro for twinning by a 180 degree rotation about rec 001.Matrix used -0.9970 -0.0004 0.0038 0.0195 -0.9997 0.0133 0.7232 0.0009 0.9991BASF refined to 0.432 (2).

### 3-Hydroxy-4-methyl-2H-thiophen-5-one (5) Fractional atomic coordinates and isotropic or equivalent isotropic displacement parameters (Å^2^)

**Table:**

	*x*	*y*	*z*	*U*_iso_*/*U*_eq_	
S1	0.6910 (2)	0.70598 (3)	0.55558 (14)	0.0183 (2)	
O1	0.8610 (6)	0.64486 (10)	0.9223 (4)	0.0231 (6)	
O2	0.0808 (6)	0.58103 (10)	0.2795 (4)	0.0203 (5)	
C1	0.6862 (8)	0.64503 (13)	0.7309 (5)	0.0164 (7)	
C2	0.4606 (8)	0.60031 (13)	0.6216 (5)	0.0164 (7)	
C3	0.3060 (8)	0.61502 (12)	0.4117 (5)	0.0155 (6)	
C4	0.3963 (8)	0.67257 (13)	0.3276 (5)	0.0160 (6)	
H4A	0.1943	0.6972	0.2798	0.019*	
H4B	0.4987	0.6669	0.1996	0.019*	
C5	0.4138 (10)	0.54539 (13)	0.7423 (6)	0.0213 (7)	
H5A	0.2293	0.5227	0.6513	0.032*	
H5B	0.3611	0.5553	0.8842	0.032*	
H5C	0.6206	0.5224	0.7701	0.032*	
H1H	0.003 (12)	0.597 (2)	0.156 (8)	0.045 (14)*	

### 3-Hydroxy-4-methyl-2H-thiophen-5-one (5) Atomic displacement parameters (Å^2^)

**Table:**

	*U*^11^	*U*^22^	*U*^33^	*U*^12^	*U*^13^	*U*^23^
S1	0.0204 (4)	0.0149 (3)	0.0183 (4)	−0.0017 (3)	0.0021 (3)	0.0002 (3)
O1	0.0287 (15)	0.0240 (11)	0.0138 (11)	0.0028 (10)	−0.0005 (10)	0.0007 (9)
O2	0.0211 (13)	0.0178 (10)	0.0187 (12)	−0.0034 (10)	−0.0017 (10)	0.0002 (9)
C1	0.0161 (17)	0.0162 (14)	0.0172 (15)	0.0052 (13)	0.0043 (12)	0.0002 (12)
C2	0.0182 (17)	0.0133 (13)	0.0189 (15)	0.0015 (12)	0.0067 (13)	0.0016 (12)
C3	0.0126 (14)	0.0138 (13)	0.0202 (16)	0.0013 (12)	0.0041 (13)	−0.0011 (12)
C4	0.0177 (16)	0.0146 (13)	0.0146 (14)	0.0001 (13)	0.0020 (12)	0.0012 (11)
C5	0.0261 (19)	0.0157 (14)	0.0226 (16)	−0.0006 (14)	0.0070 (15)	0.0044 (13)

### 3-Hydroxy-4-methyl-2H-thiophen-5-one (5) Geometric parameters (Å, º)

**Table:**

S1—C1	1.775 (3)	C2—C5	1.502 (4)
S1—C4	1.799 (3)	C3—C4	1.499 (4)
O1—C1	1.231 (4)	C4—H4A	0.9900
O2—C3	1.332 (4)	C4—H4B	0.9900
O2—H1H	0.84 (5)	C5—H5A	0.9800
C1—C2	1.440 (4)	C5—H5B	0.9800
C2—C3	1.347 (4)	C5—H5C	0.9800
			
C1—S1—C4	92.33 (15)	C3—C4—H4A	110.5
C3—O2—H1H	111 (3)	S1—C4—H4A	110.5
O1—C1—C2	127.8 (3)	C3—C4—H4B	110.5
O1—C1—S1	120.2 (3)	S1—C4—H4B	110.5
C2—C1—S1	112.0 (2)	H4A—C4—H4B	108.7
C3—C2—C1	112.2 (3)	C2—C5—H5A	109.5
C3—C2—C5	127.2 (3)	C2—C5—H5B	109.5
C1—C2—C5	120.6 (3)	H5A—C5—H5B	109.5
O2—C3—C2	123.0 (3)	C2—C5—H5C	109.5
O2—C3—C4	119.7 (3)	H5A—C5—H5C	109.5
C2—C3—C4	117.2 (3)	H5B—C5—H5C	109.5
C3—C4—S1	106.2 (2)		
			
C4—S1—C1—O1	179.5 (3)	C5—C2—C3—O2	0.3 (5)
C4—S1—C1—C2	−1.2 (3)	C1—C2—C3—C4	1.4 (4)
O1—C1—C2—C3	179.4 (3)	C5—C2—C3—C4	−179.8 (3)
S1—C1—C2—C3	0.1 (4)	O2—C3—C4—S1	177.8 (3)
O1—C1—C2—C5	0.5 (5)	C2—C3—C4—S1	−2.1 (4)
S1—C1—C2—C5	−178.8 (3)	C1—S1—C4—C3	1.7 (2)
C1—C2—C3—O2	−178.6 (3)		

### 3-Hydroxy-4-methyl-2H-thiophen-5-one (5) Hydrogen-bond geometry (Å, º)

**Table:**

*D*—H···*A*	*D*—H	H···*A*	*D*···*A*	*D*—H···*A*
O2—H1*H*···O1^i^	0.84 (5)	1.80 (5)	2.629 (3)	168 (5)
C4—H4*B*···O1^ii^	0.99	2.58	3.552 (4)	169

Symmetry codes: (i) *x*−1, *y*, *z*−1; (ii) *x*, *y*, *z*−1.
